# Combining PET/CT with serum tumor markers to improve the evaluation of histological type of suspicious lung cancers

**DOI:** 10.1371/journal.pone.0184338

**Published:** 2017-09-06

**Authors:** Rifeng Jiang, Ximin Dong, Wenzhen Zhu, Qing Duan, Yunjing Xue, Yanxia Shen, Guopeng Zhang

**Affiliations:** 1 Department of Radiology, Tongji Hospital, Tongji Medical College, Huazhong University of Science and Technology, Wuhan, China; 2 Department of Radiology, Fujian Medical University Union Hospital, Fuzhou, China; 3 Central Sterile Supply Department, Fujian Medical University Union Hospital, Fuzhou, China; 4 Department of Nuclear medicine, Tongji Hospital, Tongji Medical College, Huazhong University of Science and Technology, Wuhan, China; University of Chicago, UNITED STATES

## Abstract

**Objective:**

Histological type is important for determining the management of patients with suspicious lung cancers. In this study, PET/CT combined with serum tumor markers were used to evaluate the histological type of lung lesions.

**Materials and methods:**

Patients with suspicious lung cancers underwent ^18^F-FDG PET/CT and serum tumor markers detection. SUVmax of the tumor and serum levels of tumor markers were acquired. Differences in SUVmax and serum levels of tumor markers among different histological types of lung cancers and between EGFR mutation statues of adenocarcinoma were compared. The diagnostic efficiencies of SUVmax alone, each serum tumor marker alone, combined tumor markers and the combination of both methods were further assessed and compared.

**Results:**

SCC had the highest level of SUVmax, followed by SCLC and adenocarcinoma, and benign lesions had a lowest level. CYFRA21-1 and SCC-Ag were significantly higher in SCC, NSE was significantly higher in SCLC (P<0.001), and CEA was higher in adenocarcinoma (P = 0.343). The diagnostic efficiencies in evaluating histological types of suspicious lung cancers were insufficient when using each serum tumor marker or SUVmax alone. When combined, the AUC, sensitivity and specificity increased significantly (P<0.05 for all). Additionally, to adenocarcinoma, no significant difference was found between EGFR mutation statuses in SUVmax or serum tumor markers (P>0.05 for all).

**Conclusions:**

SUVmax and serum tumor markers show values in evaluating the histological types of suspicious lung cancers. When properly combined, the diagnostic efficiency can increase significantly.

## Introduction

Lung cancer, a malignant lung tumor, is the main cause of cancer-related deaths worldwide, with most patients present with advanced disease and poor long-term prognosis [1]. Common treatments include palliative care [2], surgery, chemotherapy, and radiation therapy [3]. Targeted therapy of lung cancer is growing in importance for advanced lung cancer [4, 5]. Histological type is important for determining management of patients with suspicious lung cancers. For therapeutic purposes, three broad classes of lung lesions are distinguished: benign lesions, non-small-cell lung carcinoma (NSCLC) and small-cell lung carcinoma (SCLC). The three main subtypes of NSCLC are adenocarcinoma, squamous-cell carcinoma (SCC) and large-cell carcinoma [3, 4]. In addition, to patients with adenocarcinoma, the mutation status of the epidermal growth factor receptor (EGFR) plays an important role in guiding the EGFR-based targeted therapy [1, 6–8].

The definitive diagnosis of suspicious lung cancer is based on histological examinations of the suspicious tissue, such as surgery or biopsy which is usually performed by bronchoscopy or CT-guidance [9]. However, it is sometimes not feasible in clinic to acquire adequate tissues for histological examination, especially for some of those patients with lung cancers in advanced-stage. Therefore, a noninvasive and accurate method for evaluating the histological type of the suspicious lesions is needed in the clinic [10].

Fuorine-18 fluorodeoxyglucose positron emission tomography (^18^F-FDG PET), a noninvasive method, has been widely used in tumor staging and therapy monitoring in patients with lung cancer [11]. FDG uptake, which reflects the glucose metabolic rate of a tumor, can be described or quantified using a metric called the maximal standard uptake value (SUVmax). Some studies have shown the value of SUVmax in evaluating the histological type of lung cancer. They demonstrated that the FDG uptake of SCC is higher than adenocarcinoma [12–14]. In addition, a higher SUVmax was associated with EGFR mutation [10].

The measurement of the concentrations of the tumor markers in serum is also a simple and feasible method to predict the histological type of a tumor [15, 16]. Previous studies have demonstrated that serum tumor markers were related to histological type with significantly higher carcino embryonie antigen (CEA) serum levels in adenocarcinomas, squmaous cell carcinoma antigen (SCC-Ag) and cytokeratin 19 fragments (CYFRA21-1) in SCC and neuron specific enolase (NSE) in SCLC [16]. Another study has further demonstrated that CEA is associated with EGFR mutation in adenocarcinomas [10].

Although SUVmax and serum tumor markers show values in evaluating the histological types of suspicious lung cancer, the role of SUVmax in evaluating other histological types of lung cancer, such as SCLC and large-cell carcinoma, is still unclear. In addition, the accuracy of these two methods alone is insufficient. To our knowledge, it remains unclear whether a combination of these two methods can result in a better diagnosis of histological types (including EGFR mutation status). Thus, the purpose of this study was to analyze these clinical and imaging parameters in suspicious lung cancers and to evaluate whether they can help predict the histological types.

## Patients and methods

### Patients

This retrospective study has been approved by the Ethics Committee of our Hospital, and abided by the statement of ethical standards, and this study was conducted between January 2016 and April 2017. Patients meeting the following inclusion criteria were enrolled: 1) Patients who were suspected of having lung cancer on the basis of conventional radiologic findings and before receiving any therapy; 2) Patients who underwent ^18^F-FDG PET/CT scan between December 2013 and January 2017 and serum tumor markers detection including CEA, CYFRA21-1, SCC-Ag and NSE; 3) Patients who underwent subsequent surgery or biopsy to confirm the histological type of the lesion. The exclusion criteria were: 1) patients who failed to confirm the histological type of the lesion in 4 weeks after ^18^F-FDG PET/CT; 2) patients with motion artifacts. We had access to information that could identify individual participants during or after data collection.

### Clinical information

All the clinical data related to the patients included in this study were recorded, and the data included age, gender, history of smoking, pre-treatment serum concentrations of CEA, CYFRA21-1, SCC-Ag and NSE, histological type and differentiation of the suspect lesion, EGFR mutation status of adenocarcinoma and the spread degree of lung cancer,. If a patient had smoked less than 100 cigarettes in his lifetime, he would be defined as never smokers, otherwise, he would be classified as smokers [10].

### Image acquisition

All patients fasted for at least 6 hours before PET/CT imaging. The serum levels of glucose were less than 150 mg/dl before an intravenous injection of 3.7MBq/kg body weight of 18F-FDG. Image acquisition started 60 min after the injection of 18F-FDG using an integrated PET/CT scanner (Discovery PET/CT Elite, GE Medical Systems, USA). The whole body PET/CT scan, usually included six to seven bed positions, was performed for all patients from the head to mid-thighs. The CT scan was performed using the following parameters: 120 kV, 28.5-150mA, 0.5s per rotation and 39.37mm per rotation. The data acquired from the CT scans were reconstructed to images with a matrix of 512 × 512 and a slice thickness of 3.75mm. PET scan was acquired in a three-dimensional imaging mode, and the acquisition time for each bed position was 90 seconds. PET datasets were reconstructed to images with a matrix of 192 × 192 using an iterative algorithm.

### PET data analysis

PET/CT data were post-processed using Advanced workstation 4.5 (GE Medical Systems, Waukesha, Wisconsin, USA). The PET data were analyzed by an experienced nuclear medicine physician who was blinded to the histological type and the EGFR mutation status, but being aware of the clinical history. A VOI was placed over the primary lung lesions to measure the SUVmax. The SUVmax was calculated as follows: SUVmax = maximum voxel activity / (injected dose / body weight) [10].

### CT image interpretation

The features of the primary lesion under CT images were measured and evaluated simultaneously by two experienced radiologists, who were blinded to the histological type and EGFR mutation status. These features included size, location and morphology of the lesions. The lesion size was the maximal diameter of the lesion. The morphological characteristics of the lesions included the density of the tumor, which can be classified as solid, ground-glass opacity (GGO) and mixed ground-glass opacity, air bronchogram, cavitation, spiculated margin and pleural tag according to the criteria described previously. If discordance in interpretation existed, two radiologists would simply resolve it by discussion and reaching a consensus [10].

### Statistical analysis

The data were analyzed using SPSS software (Version 19.0.0, IBM, Armonk, NY) and MedCalc software (https://www.medcalc.org/, version 11.4.2.0). One way Anova was used to compare the difference in SUVmax and serum level of tumor markers among different histological types, including Benign lesions, SCC, adenocarcinoma and SCLC, and Student-Newman-Keulsa was used for multiple comparisons. Binary Logistic Regression Analysis was used to combine serum tumor markers and combine SUVmax with all the serum tumor markers, therefore regression equations or models can be build, and can be expressed as a type of generalized linear model as shown in Eq 1. In the equation, p indicates the probability that a particular outcome is a case, while (1-p) indicates the probability that it is a non-case; x_m_ is the independent variables (predictors); *β*_0_ is intercept and *β*_m_ is the slope. According to the equations, prediction probabilities of different histological types of lung lesions were calculated according to regression equations [17], and the corresponding probability will be used as new markers, such as “combined tumor markers” and “SUVmax+Tumor markers”, which can be brought in the further Receiver operating characteristic (ROC) analyses. ROC curves were applied to evaluate the diagnostic efficiency of SUVmax alone, each serum tumor marker alone, combined tumor markers and combination of SUVmax and all the serum tumor markers for separating between lung cancers and benign lesions as well as NSCLC and SCLC. The Z test was applied to compare the differences in AUCs among SUVmax combined with serum tumor markers, combined tumor markers, SUVmax alone and each serum tumor markers alone. Independent-Samples T Test was used to compare the differences in SUVmax and serum level of tumor markers between different EGFR mutation statues. The alpha level was 0.05 for all the tests. All tests were two-tailed.

$ln(\left(p/\left(1\text{-}p\right)\right)=\beta_{0}+\beta_{1}x_{1}+\ldots +\beta_{m}x_{m}$**Equation 1 Generalized regression equation of the Binary Logistic Regression**.

## Results

### Patients

^18^F-FDG PET/CT were performed on 241 patients with suspected lung cancer between December 2013 and January 2017. Of the 241 patients, 33 patients were excluded because they did not undergo serum tumor markers detection, 3 patients were excluded because the histological type of the lung cancer can not be determined, and another 4 patients were excluded because they had received therapy before ^18^F-FDG PET/CT scan. Finally, a total of 201 patients were included in this study, including 32 with benign lesions (9 granuloma, 7 inflammatory change, 4 organizing pneumonia, 3 tuberculosis, 2 hamartoma, 2 pneumonomycosis, 2 sarcoidosis, 1 abscess, 1 pneumorrhagia and 1 pulmonary sequestration), 34 with SCC, 83 with adenocarcinoma, 34 with SCLC and 4 with large cell carcinoma. The clinical and pathology information for each patient was reported in S1 Table. The detail descriptive statistics for the 201 patients was shown in Table 1. The time between PET/CT or serum tumor markers detection and histological confirmation was less than 1 month. The most common symptoms were cough, expectoration, chest pain, fever, and bloody sputum.

**Table 1:** Descriptive statistics for 201 study patients.

Characteristics	Benign lesions	SCC	Adenocarcinoma	SCLC	Large-cell carcinoma
N	32	34	83	48	4
Age(years)	54.81±11.52	63.76±7.97	56.93±9.71	59.08±10.38	63.75±10.72
Gender					
Male	21	34	49	40	3
Female	11	0	34	7	1
Smoking history					
Never smokers	18	8	46	15	1
Smokers	14	26	37	33	3
Stage					
AJCC I	-	12	23	-	0
AJCC II	-	4	3	-	2
AJCC III	-	12	22	-	2
AJCC IV	-	6	35	-	0
Limited stage	-	-	-	15	-
Extensive stage	-	-	-	33	-
Acquisition of tissues					
Surgery	22	14	40	8	2
Biopsy/bronchoscope	10	20	43	40	2
Differentiation					
Well	-	2	7	0	0
Moderate	-	8	17	0	0
Poor	-	6	12	4	1
Undifferentiated	-	0	0	44	0
Indeterminate	-	18	47	0	3
EGFR status					
Wild type	-	-	8	-	-
Mutant type	-	-	8	-	-
CEA	2.33±1.55	8.39±13.57	106.80±577.64	9.89±24.23	13.71±20.87
<5	31	22	41	32	2
≧5	1	12	42	16	2
CYFRA21-1	2.13±0.95	19.15±40.38	5.73±9.09	4.07±4.42	3.13±0.93
<3.3	25	11	42	32	2
≧3.3	7	23	41	16	2
SCC-Ag	0.72±0.34	5.58±12.48	0.98±1.30	0.99±0.94	0.88±0.43
<1.5	31	15	74	43	3
≧1.5	1	19	9	5	1
NSE	11.41±3.99	15.83±6.83	14.58±7.36	61.51±75.90	19.89±19.21
<16.30	26	23	63	14	3
≧16.30	6	11	20	34	1

AJCC: American Joint Committee on Cancer, EGFR: epidermal growth factor receptor, CEA: carcino embryonie antigen, CYFRA21-1: cytokeratin 19 fragments, NSE: neuron specific enolase, SCC-Ag: squmaous cell carcinoma antigen, SCC: squamous-cell carcinoma, SCLC: small-cell lung carcinoma.

### Characteristics for suspicious lesions in PET/CT

The PET/CT imaging features of the primary lesion, including SUVmax, lesion size, location and morphology, were reported in S2 Table for each patient, and the descriptive statistics for the 201 patients were described in Table 2. SUVmax was at a high level for SCC, a middle level for adenocarcinoma, large-cell carcinoma and SCLC, and a low level for benign lesions. Larger tumor size was more often seen in patients with lung cancers than those with benign lesions. Adenocarcinoma and benign lesions more often distributed peripherally, in contrast, SCLC were more often centrally distributed. Most of patients were with lesions manifested as solid attention, and about half of the patients were with lesions with spiculated margin.

**Table 2:** PET/CT features of 201 study patients.

Characteristics	Benign lesions	SCC	Adenocarcinoma	SCLC	Large-cell carcinoma
N	32	34	83	48	4
SUVmax	6.59±5.13	14.58±5.89	10.03±4.80	12.05±4.37	11.40±5.02
Tumor size (cm)	3.34±2.52	4.74±2.22	3.68±2.03	4.86±2.37	3.18±1.34
<3	18	9	37	11	1
≧3	14	25	46	36	3
Pulmonary lobe					
RUL	9	9	22	18	0
RML	4	1	5	1	1
RLL	7	10	17	4	0
LUL	9	4	20	8	0
LLL	3	10	19	9	3
Indeterminate	0	0	0	8	0
Location					
Central	4	16	17	31	2
Peripheral	28	18	66	17	2
Morphologic pattern					
Attenuation					
GGO	0	0	1	0	0
Solid	24	27	69	46	4
Mixed	8	7	13	2	0
Internal structure					
Cavitation	6	6	13	2	0
Air bronchogram	5	2	7	0	0
Edge					
Spiculated margin	17	14	48	11	4
Nonspiculated margin	15	20	35	27	0
Pleural tag	13	8	33	1	3

RUL: right upper lobe, RML: right middle lobe, RLL: right lower lobe, LUL: left upper lobe, LLL: left lower lobe, GGO: Ground-glass opacity, SUVmax: maximal standard uptake value, SCC: squamous-cell carcinoma, SCLC: small-cell lung carcinoma.

### Differences in SUVmax and serum tumor markers among different histological types

The SUVmax and serum levels of tumor markers of different histological types were reported as mean and standard deviation in Table 3.

**Table 3:** Differences in SUVmax and serum tumor markers among different histological types.

	Category	n	CEA	CYFRA21-1	NSE	SCC-Ag	SUVmax
Benign lesions	1	32	2.331±1.548	2.127±0.953	11.409±3.991	0.722±0.342	6.592±5.129
SCLC	2	48	9.885±24.226	4.073±4.420	61.505±75.902	0.985±0.937	12.045±4.366
NSCLC	3	121	76.073±479.740	9.417±23.289	15.109±7.758	2.272±6.952	11.358±5.482
SCC	4	34	8.392±13.571	19.155±40.376	15.831±6.830	5.582±12.483	14.584±5.889
Adenocarcinoma	5	83	106.803±577.643	5.731±9.091	14.583±7.362	0.983±1.298	10.034±4.797
Large-cell carcinoma	6	4	13.705±20.867	3.130±0.925	19.890±19.205	0.875±0.427	11.400±5.019
P (ANOVA of category 1,2,3)			0.438	0.062	<0.001^a^	0.204	<0.001^a^
SNK			Category 3,2,1	Category 3,2,1	Category 2>3,1	Category 3,2,1	Category 2,3>1
P (ANOVA of category 1,2,4,5)			0.343	<0.001^a^	<0.001^a^	<0.001^a^	<0.001^a^
SNK			Category 5,2,4,1	Category 4>5,2,1	Category 2>4,5,1	Category 4>2,5,1	Category 4>2,5>1

^a^ P<0.05.

CEA: carcino embryonie antigen, CYFRA21-1: cytokeratin 19 fragments, NSE: neuron specific enolase, SCC-Ag: squmaous cell carcinoma antigen, SUVmax: maximal standard uptake value, SCLC: small-cell lung carcinoma, NSCLC: non-small-cell lung carcinoma, SCC: squamous-cell carcinoma, ANOVA: analysis of variance, SNK: Student-Newman-Keuls.

One way Anova was used to compare the difference in SUVmax and serum levels of the tumor markers among benign lesions, NSCLC and SCLC. The results were shown in Table 3 and demonstrated that SUVmax were significantly higher in lung cancers than in benign lesions (P<0.001); NSE was also found significantly higher in SCLC than in NSCLC and benign lesions (P<0.001); in contrast, CEA, CYFRA21-1 and SCC-Ag shown no significant difference among benign lesions, NSCLC and SCLC (P = 0.503).

One way Anova was further used to compare the difference in SUVmax and serum levels of the tumor markers among different histological types, including benign lesions, SCC, adenocarcinoma and SCLC. Large cell carcinomas were not included in One way Anova because the sample size was insufficient. The results were shown in Table 3 and demonstrated that SUVmax shown significant difference among above histological types (P<0.001), and it was highest for SCC, followed by NSCLC and adenocarcinoma, and lowest for benign lesions; CYFRA21-1 and SCC-Ag were significantly higher in SCC than in other histological types (P<0.001 for both); similarly, NSE was found significantly higher in SCLC (P<0.001); in contrast, CEA was at a higher level in adenocarcinoma than in other histological types, but without reaching significant (P = 0.343).

### Diagnostic efficiencies of SUVmax, each tumor marker, combined tumor markers and the combination of both methods in evaluation of histological types of lung lesions

In order to combine four serum tumor markers and combine the SUVmax with all the serum tumor markers, Binary Logistic Regression Analysis was used to build the regression equations in predicting between lung cancers and benign lesions as well as NSCLC and SCLC, as shown in Eqs 2 to 5. Therefore corresponding prediction probabilities of different histological types of lung lesions were calculated according to the four regression equations.

ln((p/(1-p))=-2.992+0.320×(CEA)+0.323×(CYFRA21-1)+0.133×(NSE)+0.791×(SCC-Ag)**Equation 2 Logistic regression equation for combing four tumor markers in predicting between lung cancers and benign lesions**.

ln((p/(1-p))=-3.939+0.298×(CEA)+0.290×(CYFRA21-1)+0.130×(NSE)+0.740×(SCC-Ag)+0.143×(SUVmax)**Equation 3 Logistic regression equation for combing SUVmax with all the serum tumor markers in predicting between lung cancers and benign lesions**.

ln((p/(1-p))=-2.431-0.012×(CEA)-0.342×(CYFRA21-1)+0.158×(NSE)-0.084×(SCC-Ag)**Equation 4 Logistic regression equation for combing four tumor markers in predicting between NSCLC and SCLC**.

ln((p/(1-p))=-2.887-0.014×(CEA)-0.359×(CYFRA21-1)+0.156×(NSE)-0.111×(SCC-Ag)+0.053×(SUVmax)**Equation 5 Logistic regression equation for combing SUVmax with all the serum tumor markers in predicting between NSCLC and SCLC**.

**Fig 1:**
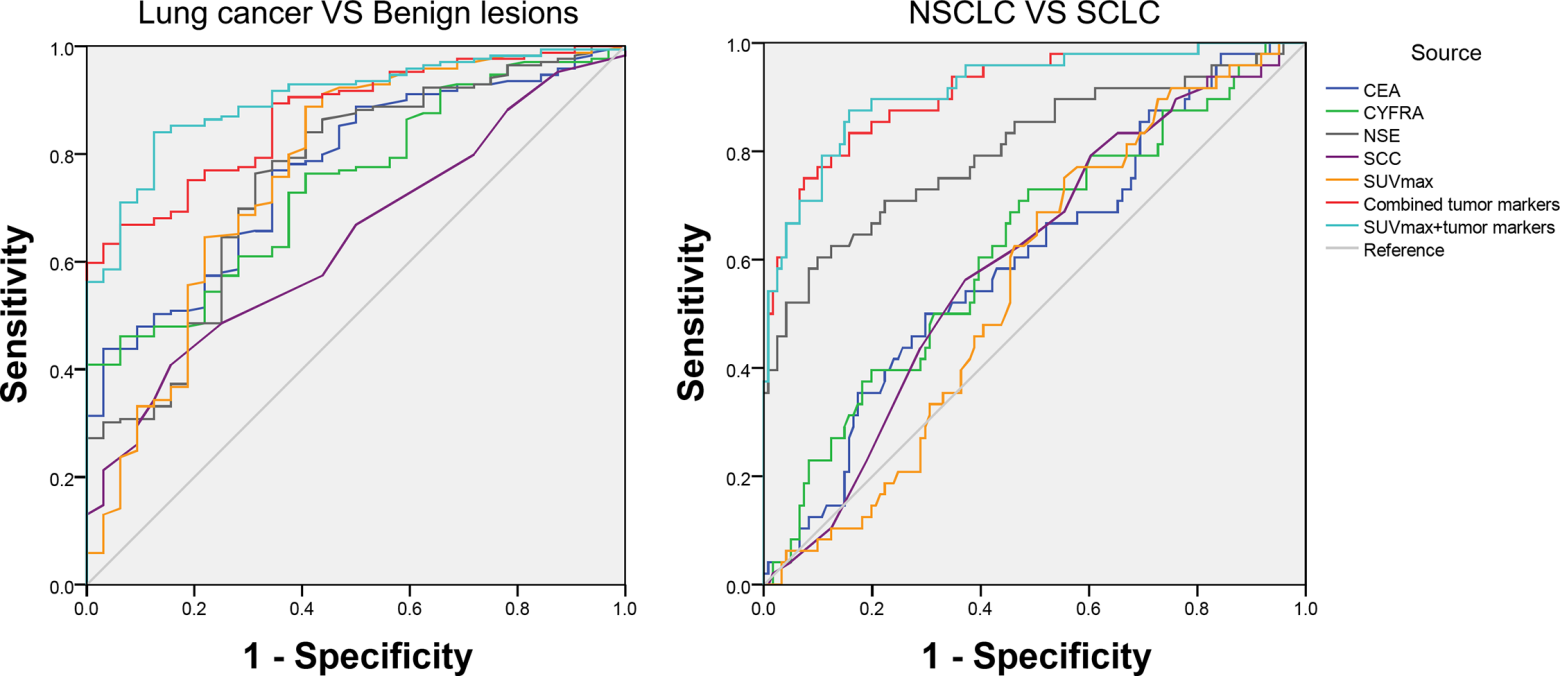
ROC curves of SUVmax, each tumor marker, combined tumor markers and the combination of both methods in evaluation of different histological types. ROC curves of SUVmax, each tumor marker, combined tumor markers and the combination of both methods in differentiating between lung cancers and benign lesions (A) and between NSCLC and SCLC (B). When SUVmax and tumor markers were combined, the AUC, sensitivity and specificity increased significantly. ROC: Receiver operating characteristic, CEA: carcino embryonie antigen, CYFRA21-1: cytokeratin 19 fragments, SCC-Ag: squmaous cell carcinoma antigen, NSE: neuron specific enolase, SUVmax: maximal standard uptake value, SCLC: small-cell lung carcinoma, NSCLC: non-small-cell lung carcinoma, AUC: areas under the curve.

ROC curve analyses were further applied to evaluate the diagnostic efficiency of SUVmax alone, each serum tumor marker alone, combined tumor markers and the prediction probability for differentiating between lung cancers and benign lesions as well as NSCLC and SCLC (Fig 1).

In differentiating between lung cancers and benign lesions, the areas under the curve (AUC) of SUVmax, CEA, CYFRA21-1, SCC-Ag and NSE were 0.767, 0.769, 0.744, 0.641 and 0.756 respectively, as shown in Table 4. SUVmax had a high sensitivity of 88.76%, but had a low specificity, in contrast, CYFRA21-1 and SCC-Ag had high specificities (100.00% and 84.38%), but had low sensitivities. When tumor markers combined, the AUC increased to 0.871 with a high specificity (93.75%) but a low sensitivity (66.86%). When SUVmax combined with all the serum tumor markers, the AUC increased to 0.902, which was significantly higher than those of SUVmax (Z = 2.357, P = 0.018), CEA (Z = 2.800, P = 0.005), CYFRA21-1 (Z = 3.326, P<0.001), SCC-Ag (Z = 4.863, P<0.001) and NSE (Z = 2.767, P = 0.006), and was higher than that of combined tumor markers (Z = 0.841, P = 0.401). The sensitivity and specificity also improved to 84.02% and 87.50% respectively.

**Table 4:** Statistical values of all metrics for differentiating between lung cancers and benign lesions and NSCLC and SCLC.

Metrics	AUC	P Value	Sensitivity	Specificity
Lung cancers VS Benign lesions				
CEA	0.769	<0.001^a^	76.92%	65.63%
CYFRA21-1	0.744	<0.001^a^	40.83%	100.00%
SCC-Ag	0.641	0.012^a^	40.83%	84.38%
NSE	0.756	<0.001^a^	76.33%	68.75%
SUVmax	0.767	<0.001^a^	88.76%	59.38%
Combined tumor markers	0.871	<0.001^a^	66.86%	93.75%
SUVmax+Tumor markers	0.902	<0.001^a^	84.02%	87.50%
NSCLC VS SCLC				
CEA	0.603	0.037^a^	50.00%	70.25%
CYFRA21-1	0.623	0.013^a^	72.92%	51.24%
SCC-Ag	0.600	0.043^a^	56.25%	62.81%
NSE	0.805	<0.001^a^	60.42%	90.08%
SUVmax	0.559	0.229	75.00%	44.63%
Combined tumor markers	0.911	<0.001^a^	75.00%	92.56%
SUVmax+Tumor markers	0.916	<0.001^a^	87.50%	84.30%

^a^ P<0.05.

AUC: area under the curve; CEA: carcino embryonie antigen, CYFRA21-1: cytokeratin 19 fragments, SCC-Ag: squmaous cell carcinoma antigen, NSE: neuron specific enolase, SUVmax: maximal standard uptake value, SCLC: small-cell lung carcinoma, NSCLC: non-small-cell lung carcinoma.

In differentiating between NSCLC and SCLC, the AUC of SUVmax, CEA, CYFRA21-1, SCC-Ag and NSE were 0.559, 0.603, 0.623, 0.600 and 0.805 respectively, as shown in Table 4. All the metrics had low sensitivities and specificities with the exception of NSE, which had a high specificity of 90.08%. When tumor markers combined, the AUC increased to 0.911 with a high specificity (92.56%) but a slightly low sensitivity (75.00%). When SUVmax combined with all the serum tumor markers, the AUC increased to 0.916, which was significantly higher than those of SUVmax (Z = 6.819, P<0.001), CEA (Z = 5.880, P<0.001), CYFRA21-1 (Z = 5.141, P<0.001), SCC-Ag (Z = 6.036, P<0.001) and NSE (Z = 2.311, P = 0.021), and was slightly higher than that of combined tumor markers (Z = 0.139, P = 0.890). The sensitivity and specificity also improved to 87.50% and 84.30% respectively.

### Differences in SUVmax and serum tumor markers between different EGFR mutation statues

Of the 83 patients with adenocarcinoma, 16 underwent additional EGFR mutation detection. EGFR mutation statuses were positive in 8 adenocarcinomas, while the other 8 were negative. The SUVmax and serum levels of tumor markers of different EGFR mutation statuses were reported as mean and standard deviation in Table 5. Independent-Samples T Test was used to compare the differences in SUVmax and serum tumor marker levels between different EGFR mutation statuses. The results demonstrated that there was no significant difference between different EGFR mutation statuses in SUVmax, CEA, CYFRA21-1, SCC-Ag or NSE (P = 0.883, 0.306, 0.713, 0.100 and 0.530 respectively).

**Table 5:** Differences in SUVmax and serum tumor markers between different EGFR mutation statues.

	EGFR mutation status		P
	Positive	Negative	
n	8	8	
CEA	708.081±1815.819	26.385±40.379	0.306
CYFRA21-1	11.174±17.408	8.560±9.175	0.713
NSE	19.626±15.111	15.834±7.048	0.530
SCC-Ag	0.650±0.450	1.163±0.689	0.100
SUVmax	9.788±3.533	10.075±4.137	0.883

CEA: carcino embryonie antigen, CYFRA21-1: cytokeratin 19 fragments, NSE: neuron specific enolase, SCC-Ag: squmaous cell carcinoma antigen, SUVmax: maximal standard uptake value, SCC: squamous-cell carcinoma, SCLC: small-cell lung carcinoma, EGFR: epidermal growth factor receptor.

## Discussion

Histological type of suspicious lung cancer is important for determining management of the disease. For therapeutic purposes, three broad classes need to be distinguished: benign lesions, NSCLC and SCLC. However, it is sometimes not feasible to acquire adequate tissues for histological examination, especially in advanced-stage patients with lung cancers [10]. Previous studies have demonstrated that both PET/CT and serum tumor markers showed values in evaluating the histological type and EGFR mutation status of lung cancer [10, 12, 13, 15, 16, 18]. In this study, noninvasive methods, PET/CT combined with serum tumor markers, were used to evaluate the histological type of the suspicious lung cancers.

The differences in SUVmax and serum tumor markers among different histological types were first compared. SUVmax was significantly higher in lung cancers than in benign lesions, however, no difference was found between NSCLC and SCLC. Further analysis shown SCC had highest level of SUVmax, followed by SCLC and adenocarcinoma, and a lowest level was found for benign lesions. CYFRA21-1 and SCC-Ag were significantly higher in SCC than in other histological types, and NSE was significantly higher in SCLC than in others. CEA was higher in adenocarcinoma than in other histological types, but without reaching significance.

Some of our results are in accord with some previous studies, which have shown that FDG uptake of SCC is higher than adenocarcinoma, because SCC displays higher glucose transporter type 1 (GLUT-1) expression than adenocarcinoma [12, 13, 18]. However, Liu et al. found that FDG PET/CT had limited diagnostic capability of predicting different histological types of lung cancer, and the reason may be due to their limited sample size, including only 15 patients with lung cancer [14]. Another study demonstrates that the FDG uptake of NSCLC is also dependent on size [18]. In our study, the average size of squamous cell carcinoma is higher than adenocarcinoma. The corresponding FDG uptake is therefore higher. Therefore, FDG uptake may be dependent both on Glut expression and size. This study additionally evaluated the FDG uptake of SCLC and benign lesions, and the FDG uptake of suspicious lesions in lung was basically divided into three levels: SCC had highest uptake of FDG, followed by SCLC and adenocarcinoma, and benign lesions shown lowest uptake. Previous study [19] has demonstrated that use of FDG-PET combined with CT was less specific in diagnosing malignancy in populations with endemic infectious lung disease compared with nonendemic regions. The average adjusted estimate of sensitivity and specificity in the characterization of malignant or benign lung nodules in regions with endemic disease were 94% and 61%, respectively. While in our study, the corresponding sensitivity and specificity are 88.76% and 59.38%, respectively, which is similar to the results of theirs. In contrast, no difference in FDG uptake was found between NSCLC and SCLC, this may be because the FDG uptake of adenocarcinoma and large-cell carcinoma lies in the same level with SCLC.

Serum tumor markers are also useful in the histological differentiation of different lung cancers. Previous studies have demonstrated that serum tumor markers were related to histological type with significantly higher CEA serum levels in adenocarcinomas, SCC and CYFRA21-1 in squamous tumors and NSE in SCLC [15, 16]. In this study, the similar results were also demonstrated. In addition, a previous study [20] investigated the association between serum tumor markers (including NSE, CEA and CYFRA21-1) and the metabolic tumor volume (MTV) or total lesion glycolysis (TLG) determined by ^18^F-FDG PET/CT in patients with recurrent SCLC. Their results demonstrated that NSE was the only tumor marker to have a strong correlation with MTV or TLG, which may serve as sensitive markers of tumor burden in patients with recurrent SCLC. Therefore, tumor markers are not only useful for the diagnosis of histological type of lung cancers, but also can indirectly reflect the tumor burden of the corresponding lung cancers, especially in advanced stages.

The diagnostic efficiencies of SUVmax alone, each serum tumor marker alone, combined tumor markers and the combination of both methods were further assessed and compared using ROC curves. It was demonstrated that using serum tumor markers or SUVmax alone, the diagnostic efficiencies were insufficient. However, when these two methods were combined, the AUC, sensitivity and specificity of evaluating histological types of lung lesions were increased vividly, and its diagnostic efficiencies were also higher than those of combined tumor markers, but not significantly. Although variable FDG uptakes or serum levels of tumor markers are correlated with histological types, one method alone is not sufficiently powerful and confident. In this study, we provided a method to combine PET/CT with serum tumor markers using Binary Logistic Regression Analysis, and this combination led to a significant increase of the diagnostic efficiency, which might be more helpful for separating different histological types of the suspicious lesion in lung, especially for differentiating between lung cancers and benign lesions as well as NSCLC and SCLC.

The detection of EGFR genotype is also important to optimize treatment in patients with lung adenocarcinoma. In this study, the rate for EGFR mutation was 50% (8/16), which is basically consistent with the results described previously [21–25]. EGFR mutations were evaluated using FDG-PET and serum levels of tumor markers. The differences in SUVmax and serum levels of tumor markers between different EGFR mutation statues were both non-significant. However, Mak et al. showed that a high FDG uptake was correlated with wild-type EGFR status, and Na et al. concluded that patients with a low SUVmax were more likely to have EGFR mutations [24, 25]. Shoji et al. were the first to reveal that EGFR mutation is related to CEA levels [26]. The possible underlying mechanism was likely to be an anti-apoptotic signal of the mutant EGFR pathway, which may elevate the expression levels of the CEA protein. The results in this study were different from previous studies, which may result from the small number of patients performing the EGFR mutation detection.

Additionally, there is increasing interest in using texture analysis for disease classification [27–29]. Texture analysis is an advanced method which can extract numerous quantitative features indicating heterogeneity of the lesion from the medical images, such as CT, MRI and PET. Different classification of disease containing different heterogeneity can be indirectly reflected by the features extracted from the medical images using texture analysis. Previous study [30] revealed that heterogeneity factor, a metric indicating intratumoral metabolic heterogeneity extracted from the PET images, was found to be statistically different between patients with adenocarcinoma and squamous-cell carcinoma who were pathologically N0. These results indicate that texture analysis has great potential in differentiating different histological types of lung cancers. Therefore, we will also use this method to differentiate different histological types of lung cancers in the future.

There were some limitations in this study. First, this study had a retrospective design and included a relatively small number of patients, especially for large-cell carcinoma. Second, a possible bias in the patient selection process may have occurred, and the cases with inadequate samples or difficult biopsy and without serum tumor marker detections were not included. Despite this limitation, the results in this study can also be used indirectly. Third, the inter-observer variability in the interpretation of CT imaging was not assessed; the observers simply reached a consensus.

## Conclusions

SUVmax and serum tumor markers show value in evaluating the histological types of lung cancer. When properly combined, the diagnostic accurate rate can increase significantly. This is helpful for determining management of the patients with suspicious lung cancers, especially in advanced-stage.

## Supporting information

S1 Table Clinical and pathology information of 201 patients with suspicious lung cancers. AJCC: American Joint Committee on Cancer, EGFR: epidermal growth factor receptor, CEA: carcino embryonie antigen, CYFRA21-1: cytokeratin 19 fragments, NSE: neuron specific enolase, SCC-Ag: squmaous cell carcinoma antigen, SCC: squamous-cell carcinoma, SCLC: small-cell lung carcinoma.
(PDF)

S2 Table Features of the primary lesions in PET/CT for 201 patients with suspicious lung cancers. RUL: right upper lobe, RML: right middle lobe, RLL: right lower lobe, LUL: left upper lobe, LLL: left lower lobe, GGO: Ground-glass opacity, SUVmax: maximal standard uptake value, SCC: squamous-cell carcinoma, SCLC: small-cell lung carcinoma.
(PDF)
