# Supplementary material for: Combining PET/CT with serum tumor markers to improve the evaluation of histological type of suspicious lung cancers
Source: PLoS One. 2017 Sep 6;12(9):e0184338. doi: 10.1371/journal.pone.0184338 (PMC5587306; doi:10.1371/journal.pone.0184338)
Supplement: S1 Table — AJCC: American Joint Committee on Cancer, EGFR: epidermal growth factor receptor, CEA: carcino embryonie antigen, CYFRA21-1: cytokeratin 19 fragments, NSE: neuron specific enolase, SCC-Ag: squmaous cell carcinoma antigen, SCC: squamous-cell carcinoma, SCLC: small-cell lung carcinoma. (PDF) [file pone.0184338.s001.pdf]

S1 Table. Clinical and pathology information of 201 patients with suspicious lung cancers

Patient	Gender	Age	CEA	CYFRA21-1	NSE	SCC-Ag	Smokers	Surgery	Biopsy	Pathology	Differentiation	EGFR mutation	Stage
1	Male	72	15.47	218.40	37.73	68.80	Yes	No	Yes	SCC	Indeterminate		AJCC stage IV
2	Female	66	7.92	2.60	13.27	1.00	No	Yes	No	SCLC	Undifferentiated		Limited stage
3	Male	80	4.07	2.35	11.29	1.90	Yes	No	Yes	SCC	Poor		AJCC stage I
4	Male	55	49.79	6.85	14.51	2.50	Yes	No	Yes	SCC	Indeterminate		AJCC stage IV
5	Male	49	4.02	4.33	13.46	0.40	Yes	Yes	No	Adenocarcinoma	Poor		AJCC stage I
6	Male	65	7.81	1.96	10.93	0.70	Yes	Yes	No	Benign lesion			
7	Male	32	0.89	1.82	11.37	11.80	Yes	Yes	No	Adenocarcinoma	Moderate		AJCC stage I
8	Male	56	3.54	10.95	11.37	1.10	Yes	Yes	No	SCC	Moderate		AJCC stage I
9	Male	68	2.06	81.74	27.44	1.90	Yes	Yes	No	SCC	Moderate		AJCC stage II
10	Male	65	6.23	4.28	12.95	0.90	Yes	No	Yes	SCC	Indeterminate		AJCC stage III
11	Male	65	15.18	4.23	13.73	2.90	Yes	No	Yes	SCC	Indeterminate		AJCC stage IV
12	Female	70	1.43	3.58	10.41	0.40	No	Yes	No	Benign lesion			
13	Male	63	9.66	7.11	18.89	2.10	Yes	No	Yes	SCC	Indeterminate		AJCC stage III
14	Female	62	215.84	3.86	22.81	0.50	No	No	Yes	Adenocarcinoma	Indeterminate		AJCC stage IV
15	Male	81	5.39	5.43	7.57	0.70	Yes	No	Yes	SCC	Indeterminate		AJCC stage II
16	Male	65	22.86	3.59	7.83	0.90	Yes	No	Yes	Adenocarcinoma	Indeterminate		AJCC stage IV
17	Male	73	1.73	3.67	8.45	1.50	Yes	Yes	No	Adenocarcinoma	Moderate		AJCC stage III
18	Female	45	26.85	2.18	26.65	0.50	No	No	Yes	Adenocarcinoma	Poor		AJCC stage IV
19	Male	45	5.45	6.46	14.32	0.80	No	Yes	No	Adenocarcinoma	Indeterminate		AJCC stage IV
20	Male	50	761.93	5.56	19.65	0.60	No	No	Yes	Adenocarcinoma	Indeterminate		AJCC stage IV
21	Male	56	4.54	2.86	65.14	1.00	Yes	No	Yes	SCLC	Undifferentiated		Extensive stage
22	Male	57	115.67	59.80	46.98	0.60	No	No	Yes	Adenocarcinoma	Indeterminate		AJCC stage IV
23	Male	58	3.45	2.29	9.93	0.50	No	No	Yes	Adenocarcinoma	Indeterminate		AJCC stage III

24	Female	61	4.46	1.77	10.83	0.40	No	No	No	Adenocarcinoma	Indeterminate		AJCC stage IV
25	Male	49	1.88	1.70	12.33	0.40	Yes	Yes	No	Benign lesion			
26	Female	51	3.23	1.60	19.23	0.30	No	Yes	No	Adenocarcinoma	Well		AJCC stage I
27	Female	55	3.31	2.48	8.98	0.60	No	No	Yes	Benign lesion			
28	Female	57	23.13	6.78	11.07	1.20	No	Yes	No	Adenocarcinoma	Indeterminate		AJCC stage I
29	Male	49	4.21	3.22	58.31	0.60	No	No	No	SCLC	Undifferentiated		Extensive stage
30	Male	72	1.45	1.97	8.84	0.80	No	Yes	No	Adenocarcinoma	Well		AJCC stage I
31	Male	69	0.98	3.39	10.03	0.60	No	Yes	No	Benign lesion			
32	Female	53	13.23	2.54	11.49	1.30	No	No	Yes	Adenocarcinoma	Indeterminate	Negative	AJCC stage IV
33	Male	58	2.76	0.94	16.59	1.10	No	Yes	No	Benign lesion			
34	Female	41	1.92	3.03	12.14	0.70	No	No	No	Adenocarcinoma	Indeterminate		AJCC stage I
35	Male	72	3.82	3.56	14.17	0.80	Yes	Yes	No	Benign lesion			
36	Male	56	4.68	1.69	9.10	1.90	Yes	Yes	No	Benign lesion			
37	Male	36	1.26	1.15	9.27	0.60	No	Yes	No	Benign lesion			
38	Female	70	2.72	2.98	11.24	0.80	No	No	No	Adenocarcinoma	Moderate	Positive	AJCC stage II
39	Male	36	0.70	0.74	8.34	0.50	No	No	Yes	Benign lesion			
40	Male	65	364.69	7.61	27.27	0.60	Yes	No	Yes	Adenocarcinoma	Poor		AJCC stage IV
41	Female	76	269.43	5.27	17.56	0.90	No	No	Yes	Adenocarcinoma	Indeterminate		AJCC stage III
42	Male	63	3.01	2.82	18.09	3.00	No	No	Yes	SCC	Indeterminate		AJCC stage IV
43	Male	51	1.67	2.51	11.24	0.90	Yes	Yes	No	Benign lesion			
44	Male	51	3.39	2.23	12.06	0.80	Yes	Yes	No	Adenocarcinoma	Indeterminate	Negative	AJCC stage I
45	Female	60	1.88	2.91	12.92	0.60	No	Yes	No	Adenocarcinoma	Poor		AJCC stage III
46	Female	54	2.64	1.21	12.91	0.50	No	Yes	No	Adenocarcinoma	Moderate		AJCC stage I
47	Female	60	1.54	2.32	22.89	0.90	No	No	No	SCLC	Undifferentiated		Extensive stage
48	Male	66	82.74	4.14	9.43	1.20	Yes	Yes	No	Adenocarcinoma	Indeterminate		AJCC stage III

49	Male	52	2.44	1.16	21.46	0.60	Yes	No	Yes	Adenocarcinoma	Indeterminate		AJCC stage IV
50	Female	47	0.90	3.24	10.55	0.70	No	Yes	No	Adenocarcinoma	Moderate		AJCC stage I
51	Male	62	5.66	47.60	22.53	14.70	No	No	Yes	SCC	Indeterminate		AJCC stage III
52	Female	70	4.27	1.86	12.63	0.50	No	No	Yes	Adenocarcinoma	Indeterminate		AJCC stage IV
53	Female	66	1.48	2.90	18.80	1.00	No	Yes	No	Benign lesion			
54	Male	76	1.11	3.76	48.39	0.60	Yes	No	Yes	Large-cell carcinoma	Indeterminate		AJCC stage II
55	Male	48	1.51	2.40	21.39	0.30	Yes	Yes	No	Benign lesion			
56	Male	43	16.83	10.64	11.63	0.90	No	No	Yes	Adenocarcinoma	Indeterminate		AJCC stage IV
57	Female	47	228.48	12.11	16.21	0.20	No	No	Yes	Adenocarcinoma	Indeterminate	Positive	AJCC stage IV
58	Male	69	47.23	5.75	13.88	1.50	Yes	No	Yes	Adenocarcinoma	Indeterminate		AJCC stage III
59	Male	72	26.78	3.44	15.15	0.90	Yes	No	Yes	Adenocarcinoma	Indeterminate		AJCC stage IV
60	Male	63	2.88	1.80	10.09	1.50	Yes	Yes	No	Large-cell carcinoma	Indeterminate		AJCC stage II
61	Male	61	57.62	12.47	16.79	3.70	Yes	No	Yes	SCC	Indeterminate		AJCC stage II
62	Male	77	34.10	11.98	53.76	4.80	Yes	No	Yes	SCLC	Poor		Extensive stage
63	Male	55	1.60	1.08	14.16	0.30	No	No	Yes	Benign lesion			
64	Male	77	2.54	4.28	9.67	1.30	Yes	Yes	No	SCC	Moderate		AJCC stage I
65	Male	48	2.66	6.90	12.11	0.80	Yes	Yes	No	SCC	Moderate		AJCC stage I
66	Female	52	17.90	2.41	11.45	0.60	No	No	Yes	Adenocarcinoma	Indeterminate		AJCC stage I
67	Female	58	2.57	3.82	14.04	0.40	No	Yes	No	Adenocarcinoma	Poor		AJCC stage III
68	Male	52	2.33	16.93	22.45	4.60	Yes	Yes	No	SCC	Moderate		AJCC stage III
69	Male	66	5.97	3.20	7.12	0.60	Yes	Yes	No	Large-cell carcinoma	Poor		AJCC stage III
70	Female	54	3.50	1.94	10.17	0.80	No	No	Yes	SCLC	Undifferentiated		Limited stage
71	Male	66	5.38	1.70	12.95	0.70	Yes	Yes	No	SCC	Poor		AJCC stage I
72	Male	66	57.47	29.53	16.28	0.70	Yes	No	Yes	Adenocarcinoma	Poor	Negative	AJCC stage III
73	Female	56	0.84	1.70	10.03	0.60	No	Yes	No	Adenocarcinoma	Indeterminate		AJCC stage I

74	Male	44	1.60	0.78	15.41	1.10	Yes	No	Yes	Adenocarcinoma	Poor		AJCC stage IV
75	Male	68	8.12	6.67	11.99	0.90	No	No	Yes	Adenocarcinoma	Indeterminate		AJCC stage III
76	Male	58	163.06	4.37	40.14	0.40	Yes	No	Yes	SCLC	Undifferentiated		Extensive stage
77	Female	48	1.71	1.76	8.52	1.60	No	Yes	No	Adenocarcinoma	Well		AJCC stage I
78	Male	55	3.67	5.78	27.56	1.20	Yes	Yes	No	Adenocarcinoma	Moderate	Negative	AJCC stage III
79	Female	70	11.43	5.72	19.55	1.90	No	Yes	No	Adenocarcinoma	Well		AJCC stage I
80	Male	51	4.91	2.57	12.39	0.60	Yes	Yes	No	Adenocarcinoma	Moderate		AJCC stage I
81	Female	49	52.17	8.08	13.22	0.40	No	No	Yes	Adenocarcinoma	Indeterminate		AJCC stage IV
82	Female	58	1.12	1.88	9.82	0.60	No	Yes	No	Benign lesion			
83	Male	60	4.14	4.04	9.76	1.30	Yes	Yes	No	Benign lesion			
84	Male	58	4.83	1.16	8.32	0.60	No	Yes	No	Benign lesion			
85	Male	60	2.60	1.84	11.41	2.10	Yes	Yes	No	SCC	Moderate		AJCC stage I
86	Male	67	1.71	2.07	14.44	1.10	No	Yes	No	SCC	Moderate		AJCC stage I
87	Male	64	21.27	2.17	11.64	1.00	Yes	No	Yes	Adenocarcinoma	Indeterminate		AJCC stage IV
88	Male	47	0.71	2.14	19.22	0.80	Yes	Yes	No	Benign lesion			
89	Male	66	8.59	5.98	77.50	0.70	Yes	No	Yes	SCLC	Undifferentiated		Extensive stage
90	Male	46	4.05	7.00	16.32	0.70	Yes	No	Yes	Adenocarcinoma	Indeterminate		AJCC stage IV
91	Female	42	0.83	1.05	6.84	0.60	No	No	Yes	Benign lesion			
92	Male	61	1.24	1.92	7.96	0.90	Yes	Yes	No	Benign lesion			
93	Male	46	2.07	1.34	8.84	0.80	Yes	No	Yes	Benign lesion			
94	Female	75	3.71	2.66	11.90	0.90	No	Yes	No	Benign lesion			
95	Female	51	0.68	1.80	27.96	0.40	No	No	Yes	SCLC	Poor		Extensive stage
96	Male	59	1.98	2.75	370.00	0.40	Yes	No	Yes	SCLC	Undifferentiated		Extensive stage
97	Male	61	1.57	1.97	17.27	1.00	Yes	Yes	No	SCC	Poor		AJCC stage III
98	Male	66	5.23	3.11	10.49	0.80	Yes	Yes	No	Adenocarcinoma	Indeterminate		AJCC stage I

99	Female	55	4.84	13.32	17.60	2.70	No	No	Yes	Adenocarcinoma	Indeterminate	Negative	AJCC stage IV
100	Male	60	5.74	5.17	10.02	1.00	Yes	Yes	No	Adenocarcinoma	Moderate		AJCC stage II
101	Male	65	203.95	4.84	9.57	1.50	Yes	Yes	No	Adenocarcinoma	Indeterminate	Positive	AJCC stage III
102	Male	74	2.58	1.71	10.72	1.00	Yes	Yes	No	Adenocarcinoma	Moderate		AJCC stage I
103	Male	70	2.14	3.52	17.23	0.60	Yes	No	Yes	Adenocarcinoma	Indeterminate		AJCC stage III
104	Male	57	6.94	2.39	12.23	1.10	Yes	Yes	No	Adenocarcinoma	Well		AJCC stage I
105	Male	38	3.67	1.96	9.04	0.80	No	No	Yes	Benign lesion			
106	Female	56	16.48	2.64	13.82	0.50	No	Yes	No	Adenocarcinoma	Well	Positive	AJCC stage IV
107	Male	50	6.38	2.53	12.73	1.30	Yes	Yes	No	Adenocarcinoma	Moderate		AJCC stage III
108	Male	20	1.89	3.34	14.96	1.30	No	No	Yes	SCLC	Undifferentiated		Extensive stage
109	Female	56	1.28	1.83	9.94	0.60	No	Yes	No	Benign lesion			
110	Male	69	3.53	4.87	8.91	0.70	Yes	No	Yes	Adenocarcinoma	Indeterminate		AJCC stage III
111	Male	49	3.60	8.16	10.94	1.30	No	No	Yes	Adenocarcinoma	Poor		AJCC stage IV
112	Male	60	115.43	5.90	8.87	1.10	Yes	Yes	No	Adenocarcinoma	Poor	Negative	AJCC stage III
113	Male	58	1.85	1.19	9.46	0.90	Yes	Yes	No	Adenocarcinoma	Moderate		AJCC stage I
114	Female	37	5.18	0.67	11.40	0.80	No	Yes	No	Adenocarcinoma	Moderate		AJCC stage I
115	Male	65	1.64	4.08	12.27	2.40	Yes	Yes	No	SCC	Well		AJCC stage I
116	Female	65	1.72	2.45	12.85	0.30	No	Yes	No	Adenocarcinoma	Well		AJCC stage I
117	Female	46	1.32	1.46	8.21	0.70	No	Yes	No	Benign lesion			
118	Male	63	2.96	4.33	19.91	1.20	Yes	No	Yes	Adenocarcinoma	Indeterminate		AJCC stage IV
119	Female	53	7.84	1.54	16.58	0.30	No	No	Yes	Adenocarcinoma	Moderate		AJCC stage IV
120	Male	67	7.34	9.14	14.28	27.20	Yes	No	Yes	SCC	Indeterminate		AJCC stage IV
121	Male	61	3.10	9.15	27.28	0.40	Yes	Yes	No	Adenocarcinoma	Indeterminate	Positive	AJCC stage IV
122	Male	67	3.40	13.11	12.83	2.00	Yes	No	Yes	SCC	Indeterminate		AJCC stage III
123	Male	61	2.11	5.15	10.47	2.20	Yes	No	Yes	SCC	Indeterminate		AJCC stage III

124	Male	66	2.31	5.50	17.02	1.00	Yes	Yes	No	Adenocarcinoma	Moderate		AJCC stage IV
125	Male	61	573.81	3.90	9.38	0.80	No	No	Yes	Adenocarcinoma	Indeterminate		AJCC stage III
126	Female	65	4.29	2.45	11.12	0.90	No	No	Yes	Adenocarcinoma	Indeterminate		AJCC stage IV
127	Male	37	1.14	0.98	9.52	0.40	No	No	Yes	Adenocarcinoma	Indeterminate		AJCC stage I
128	Female	50	59.39	2.26	15.73	0.40	No	Yes	No	Adenocarcinoma	Moderate		AJCC stage III
129	Male	54	9.26	0.82	10.56	0.50	Yes	No	Yes	SCLC	Undifferentiated		Extensive stage
130	Male	61	1.30	3.38	9.78	0.80	Yes	Yes	No	Benign lesion			
131	Female	58	33.83	5.10	16.34	0.70	No	No	Yes	Adenocarcinoma	Indeterminate		AJCC stage IV
132	Female	51	2.09	2.10	8.04	1.30	No	Yes	No	Adenocarcinoma	Moderate		AJCC stage III
133	Male	49	3.22	54.01	24.44	4.50	Yes	No	Yes	SCC	Indeterminate		AJCC stage III
134	Male	51	1.68	7.74	61.25	0.80	No	No	Yes	SCLC	Undifferentiated		Extensive stage
135	Male	67	41.39	2.52	14.63	1.00	No	No	Yes	SCC	Indeterminate		AJCC stage III
136	Female	63	1.73	1.88	10.96	0.40	No	Yes	No	Benign lesion			
137	Female	51	47.58	2.08	10.18	0.50	No	No	Yes	Adenocarcinoma	Indeterminate		AJCC stage IV
138	Male	63	3.35	1.73	10.27	0.40	No	No	Yes	SCC	Indeterminate		AJCC stage I
139	Male	47	1.68	6.04	24.30	0.40	Yes	No	Yes	Adenocarcinoma	Indeterminate	Negative	AJCC stage III
140	Male	74	5195.9	53.30	54.44	0.90	Yes	No	Yes	Adenocarcinoma	Poor	Positive	AJCC stage IV
141	Female	48	5.56	5.95	11.43	0.30	No	No	Yes	Adenocarcinoma	Indeterminate		AJCC stage IV
142	Male	56	13.21	2.19	13.04	0.80	Yes	No	Yes	Adenocarcinoma	Indeterminate	Positive	AJCC stage IV
143	Male	67	2.45	1.73	13.75	16.20	Yes	No	Yes	SCC	Indeterminate		AJCC stage III
144	Male	55	2.87	4.73	15.03	0.80	No	No	Yes	Adenocarcinoma	Indeterminate		AJCC stage IV
145	Male	70	3.41	1.40	11.21	1.20	No	Yes	No	SCC	Poor		AJCC stage II
146	Male	48	11.37	3.14	8.51	1.10	Yes	No	Yes	Adenocarcinoma	Poor	Negative	AJCC stage III
147	Female	46	2.01	1.07	10.09	0.30	No	Yes	No	Benign lesion			
148	Male	69	3.38	3.84	7.86	0.90	Yes	Yes	No	SCC	Poor		AJCC stage I

149	Female	53	3.13	21.62	13.76	0.50	No	Yes	No	Adenocarcinoma	Poor		AJCC stage III
150	Male	48	1.39	1.43	11.99	0.70	Yes	No	Yes	Adenocarcinoma	Indeterminate		AJCC stage II
151	Male	33	3.37	1.49	18.16	0.50	Yes	No	Yes	Benign lesion			
152	Male	66	5.40	2.38	14.51	1.20	Yes	No	Yes	SCC	Indeterminate		AJCC stage I
153	Male	67	1.21	2.24	12.70	0.70	Yes	Yes	No	Adenocarcinoma	Indeterminate		AJCC stage I
154	Female	66	1.28	1.50	8.22	0.60	No	Yes	No	SCLC	Undifferentiated		Extensive stage
155	Male	66	7.64	5.63	10.30	3.20	Yes	No	Yes	Adenocarcinoma	Indeterminate		AJCC stage III
156	Male	45	23.77	7.57	15.67	0.60	No	No	Yes	Adenocarcinoma	Indeterminate		AJCC stage IV
157	Male	77	2.75	3.93	17.33	1.30	Yes	No	Yes	Benign lesion			
158	Male	64	0.71	7.51	12.04	11.10	No	No	Yes	SCC	Indeterminate		AJCC stage III
159	Male	46	4.24	8.56	17.15	1.50	Yes	Yes	No	SCC	Well		AJCC stage III
160	Female	50	1.64	1.44	5.34	0.30	No	No	Yes	Benign lesion			
161	Male	71	4.51	1.75	14.08	0.70	Yes	Yes	No	SCLC	Undifferentiated		Limited stage
162	Male	51	1.97	3.34	7.83	0.80	Yes	No	Yes	Benign lesion			
163	Male	58	3.08	68.11	34.45	0.80	Yes	No	Yes	SCC	Poor		AJCC stage IV
164	Female	54	17.66	2.25	10.79	1.50	No	No	Yes	Adenocarcinoma	Indeterminate		AJCC stage IV
165	Female	51	0.83	2.18	11.41	0.10	No	Yes	No	Adenocarcinoma	Moderate	Positive	AJCC stage IV
166	Male	67	3.73	28.07	14.89	1.40	No	Yes	No	SCC	Moderate		AJCC stage I
167	Male	66	1.33	3.71	13.70	0.50	Yes	No	Yes	SCLC	Poor		Limited stage
168	Male	61	5.42	1.59	83.94	0.80	Yes	No	No	SCLC	Undifferentiated		Extensive stage
169	Male	64	12.17	1.90	22.07	0.60	Yes	No	Yes	SCLC	Undifferentiated		Extensive stage
170	Male	63	42.84	25.80	263.70	3.10	Yes	No	Yes	SCLC	Undifferentiated		Extensive stage
171	Male	63	10.35	6.17	28.50	0.60	Yes	No	Yes	SCLC	Undifferentiated		Extensive stage
172	Male	67	3.31	1.79	12.48	4.90	Yes	No	Yes	SCLC	Undifferentiated		Limited stage
173	Male	53	3.10	2.38	46.01	0.70	Yes	No	Yes	SCLC	Undifferentiated		Extensive stage

174	Male	47	7.00	2.01	244.10	1.00	Yes	No	Yes	SCLC	Undifferentiated		Limited stage
175	Male	64	2.57	1.49	15.92	0.90	No	Yes	No	SCLC	Undifferentiated		Limited stage
176	Male	74	2.62	6.15	104.70	0.60	Yes	No	Yes	SCLC	Undifferentiated		Extensive stage
177	Male	62	7.47	2.36	14.01	1.00	Yes	No	Yes	SCLC	Undifferentiated		Extensive stage
178	Male	69	3.96	2.71	9.03	1.30	No	No	No	SCLC	Poor		Limited stage
179	Male	42	2.33	1.29	81.02	0.50	Yes	No	Yes	SCLC	Undifferentiated		Extensive stage
180	Male	58	2.76	1.37	12.41	0.50	Yes	No	Yes	SCLC	Undifferentiated		Limited stage
181	Male	46	1.07	3.23	19.23	0.90	Yes	No	No	SCLC	Undifferentiated		Limited stage
182	Male	63	2.05	1.63	38.73	0.50	Yes	No	Yes	SCLC	Undifferentiated		Extensive stage
183	Male	63	1.88	1.57	17.50	1.10	Yes	No	Yes	SCLC	Undifferentiated		Limited stage
184	Female	63	1.86	0.90	12.97	0.60	No	No	Yes	SCLC	Undifferentiated		Limited stage
185	Male	57	10.96	3.53	149.40	1.50	Yes	No	Yes	SCLC	Undifferentiated		Extensive stage
186	Male	68	1.86	2.28	17.03	0.60	Yes	No	Yes	SCLC	Undifferentiated		Limited stage
187	Female	50	44.86	3.76	13.96	0.80	No	No	Yes	Large-cell carcinoma	Indeterminate		AJCC stage III
188	Male	69	3.53	15.20	72.34	0.70	Yes	No	Yes	SCLC	Undifferentiated		Extensive stage
189	Male	54	22.57	1.84	25.42	0.50	Yes	No	Yes	SCLC	Undifferentiated		Extensive stage
190	Male	51	27.57	6.24	101.00	0.70	Yes	No	Yes	SCLC	Undifferentiated		Extensive stage
191	Male	69	2.45	2.87	53.34	1.40	Yes	No	Yes	SCLC	Undifferentiated		Extensive stage
192	Male	53	19.14	4.73	73.90	0.30	No	No	Yes	SCLC	Undifferentiated		Extensive stage
193	Male	41	10.38	1.75	25.10	1.10	No	Yes	No	SCLC	Undifferentiated		Extensive stage
194	Male	72	1.75	3.07	31.51	1.00	No	No	No	SCLC	Undifferentiated		Extensive stage
195	Female	57	0.82	1.51	73.22	0.20	No	No	Yes	SCLC	Undifferentiated		Limited stage
196	Male	48	1.77	4.51	211.90	0.70	Yes	No	Yes	SCLC	Undifferentiated		Extensive stage
197	Male	48	1.80	10.03	175.90	0.60	Yes	No	Yes	SCLC	Undifferentiated		Extensive stage
198	Male	63	2.82	2.36	24.81	1.80	Yes	Yes	No	SCLC	Undifferentiated		Extensive stage

199	Male	68	2.38	10.90	11.96	0.60	Yes	No	Yes	SCLC	Undifferentiated		Extensive stage
200	Male	72	1.87	1.65	17.19	0.60	Yes	Yes	No	SCLC	Undifferentiated		Limited stage
201	Male	66	6.29	1.78	10.70	1.30	Yes	Yes	No	Adenocarcinoma	Indeterminate		AJCC stage I

AJCC: American Joint Committee on Cancer, EGFR: epidermal growth factor receptor, CEA: carcino embryonie antigen, CYFRA21-1: cytokeratin 19 fragments, NSE: neuron specific enolase, SCC-Ag: squmaous cell carcinoma antigen, SCC: squamous-cell carcinoma, SCLC: small-cell lung carcinoma.
