# Supplementary material for: Combining PET/CT with serum tumor markers to improve the evaluation of histological type of suspicious lung cancers
Source: PLoS One. 2017 Sep 6;12(9):e0184338. doi: 10.1371/journal.pone.0184338 (PMC5587306; doi:10.1371/journal.pone.0184338)
Supplement: S2 Table — RUL: right upper lobe, RML: right middle lobe, RLL: right lower lobe, LUL: left upper lobe, LLL: left lower lobe, GGO: Ground-glass opacity, SUVmax: maximal standard uptake value, SCC: squamous-cell carcinoma, SCLC: small-cell lung carcinoma. (PDF) [file pone.0184338.s002.pdf]

**S2 Table. Features of the primary mass in PET/CT for 201 patients with suspicious lung cancers**

| Patient | Pathology | SUVmax | Size | Location of primary lesion | Central location | Density | Cavitation | Air bronchogram | Spiculated margin | Pleural tag |
|---|---|---|---|---|---|---|---|---|---|---|
| 1 | SCC | 13.72 | 3.8 | RLL | | Solid | | | | |
| 2 | SCLC | 5.64 | 1 | RUL | | Solid | | | | |
| 3 | SCC | 17.72 | 3.2 | LUL | | Solid | | | | Yes |
| 4 | SCC | 20.93 | 5.6 | LUL | Yes | Solid | | Yes | | |
| 5 | Adenocarcinoma | 12.82 | 6 | RUL | | Mixed | Yes | | Yes | |
| 6 | Benign lesion | 7.80 | 2.6 | RUL | | Mixed | | | Yes | |
| 7 | Adenocarcinoma | 7.29 | 1 | RUL | | Solid | | | Yes | |
| 8 | SCC | 17.84 | 6 | LLL | | Mixed | Yes | | Yes | |
| 9 | SCC | 25.17 | 4.3 | RUL | Yes | Mixed | Yes | | Yes | |
| 10 | SCC | 7.10 | 1.3 | RUL | | Mixed | | | Yes | |
| 11 | SCC | 11.87 | 5.7 | LLL | | Solid | | | | |
| 12 | Benign lesion | 4.64 | 0.9 | LUL | | Solid | | | Yes | |
| 13 | SCC | 16.89 | 3.2 | RLL | | Solid | | | Yes | |
| 14 | Adenocarcinoma | 15.68 | 3.2 | LLL | Yes | Solid | | | Yes | |
| 15 | SCC | 11.10 | 8.2 | RLL | | Mixed | Yes | | Yes | Yes |
| 16 | Adenocarcinoma | 10.68 | 2.5 | LUL | | Solid | | | Yes | Yes |
| 17 | Adenocarcinoma | 9.00 | 3.6 | RLL | | Solid | | | Yes | Yes |
| 18 | Adenocarcinoma | 8.37 | 3.1 | LLL | | Solid | | | Yes | |
| 19 | Adenocarcinoma | 4.14 | 1.6 | LLL | | Solid | | | | Yes |
| 20 | Adenocarcinoma | 5.20 | 1.8 | LLL | | Mixed | Yes | | Yes | Yes |
| 21 | SCLC | 10.40 | 5.3 | RUL | Yes | Solid | | | | |

| | | | | | | | | | | |
|---|---|---|---|---|---|---|---|---|---|---|
| 22 | Adenocarcinoma | 19.10 | 10.2 | LUL | Yes | Solid | Yes | | Yes | |
| 23 | Adenocarcinoma | 16.80 | 3 | LLL | | Solid | | | Yes | Yes |
| 24 | Adenocarcinoma | 18.50 | 6 | LUL | Yes | Solid | Yes | | | |
| 25 | Benign lesion | 13.50 | 8.4 | RUL | | Mixed | Yes | | Yes | |
| 26 | Adenocarcinoma | 9.16 | 2.7 | RUL | | Mixed | | | Yes | |
| 27 | Benign lesion | 5.40 | 3.7 | RML | | Solid | | | | |
| 28 | Adenocarcinoma | 9.90 | 7.2 | LLL | | Mixed | Yes | | Yes | Yes |
| 29 | SCLC | 8.20 | 4.7 | RUL | Yes | Solid | | | | |
| 30 | Adenocarcinoma | 13.16 | 3.8 | LUL | | Solid | | | Yes | Yes |
| 31 | Benign lesion | 2.30 | 1.5 | RLL | | Solid | | | Yes | Yes |
| 32 | Adenocarcinoma | 9.00 | 2.5 | LLL | | Solid | | | Yes | Yes |
| 33 | Benign lesion | 19.90 | 9.7 | RML | Yes | Solid | | | | |
| 34 | Adenocarcinoma | 7.10 | 2 | LUL | | Solid | | | Yes | |
| 35 | Benign lesion | 8.50 | 4.8 | RUL | | Solid | | | Yes | |
| 36 | Benign lesion | 2.70 | 1.8 | LLL | | Solid | | | Yes | Yes |
| 37 | Benign lesion | 4.10 | 2.5 | LUL | Yes | Mixed | | Yes | Yes | |
| 38 | Adenocarcinoma | 12.80 | 3.6 | RLL | | Solid | | | Yes | |
| 39 | Benign lesion | 8.90 | 10.2 | LUL | | Solid | Yes | | | |
| 40 | Adenocarcinoma | 11.30 | 5.1 | LUL | Yes | Solid | | | Yes | Yes |
| 41 | Adenocarcinoma | 15.50 | 6.5 | RLL | | Solid | | | Yes | |
| 42 | SCC | 14.60 | 5 | RUL | Yes | Solid | | | Yes | |
| 43 | Benign lesion | 0.60 | 8.3 | RML | | Solid | Yes | | Yes | |
| 44 | Adenocarcinoma | 5.30 | 4.2 | RLL | Yes | Solid | | | | |
| 45 | Adenocarcinoma | 8.40 | 2.5 | RLL | | Solid | | | Yes | Yes |
| 46 | Adenocarcinoma | 2.40 | 1.8 | RUL | | Solid | | | Yes | Yes |

| | | | | | | | | | | |
|---|---|---|---|---|---|---|---|---|---|---|
| 47 | SCLC | 4.50 | 2.1 | LLL | | Solid | | | Yes | |
| 48 | Adenocarcinoma | 9.90 | 2.6 | RML | | Solid | | | Yes | |
| 49 | Adenocarcinoma | 10.30 | 5.8 | LUL | Yes | Solid | | | Yes | |
| 50 | Adenocarcinoma | 7.50 | 2.4 | RLL | | Mixed | Yes | | Yes | |
| 51 | SCC | 10.50 | 5.1 | RUL | | Solid | | | Yes | |
| 52 | Adenocarcinoma | 6.60 | 2.7 | LUL | | Solid | | | Yes | Yes |
| 53 | Benign lesion | 3.80 | 4.8 | LUL | | Mixed | | Yes | | |
| 54 | Large-cell carcinoma | 12.10 | 4 | LLL | | Solid | | | Yes | Yes |
| 55 | Benign lesion | 1.70 | 1 | RUL | | Solid | | | Yes | Yes |
| 56 | Adenocarcinoma | 9.90 | 4.3 | LUL | | Solid | | | Yes | Yes |
| 57 | Adenocarcinoma | 11.30 | 6.8 | LUL | | Solid | | | Yes | |
| 58 | Adenocarcinoma | 18.20 | 3.2 | RML | | Solid | | | Yes | Yes |
| 59 | Adenocarcinoma | 17.70 | 5.4 | RUL | | Solid | | | Yes | |
| 60 | Large-cell carcinoma | 14.60 | 3.5 | LLL | Yes | Solid | | | Yes | |
| 61 | SCC | 19.40 | 2.8 | RLL | | Mixed | Yes | | | Yes |
| 62 | SCLC | 13.90 | 6.2 | LUL | | Solid | | | | |
| 63 | Benign lesion | 9.70 | 2 | LUL | | Solid | | | Yes | Yes |
| 64 | SCC | 7.90 | 6.1 | RML | Yes | Solid | | | Yes | |
| 65 | SCC | 15.10 | 7.4 | LLL | | Mixed | Yes | | Yes | |
| 66 | Adenocarcinoma | 7.20 | 4.9 | LUL | | Mixed | Yes | | Yes | |
| 67 | Adenocarcinoma | 10.00 | 2.1 | LLL | | Solid | | | Yes | |
| 68 | SCC | 14.40 | 6 | RUL | Yes | Solid | | | | |
| 69 | Large-cell carcinoma | 14.80 | 4 | LLL | | Solid | | | Yes | Yes |
| 70 | SCLC | 7.60 | 5.2 | LUL | Yes | Solid | | | | |
| 71 | SCC | 12.00 | 2.6 | RUL | | Solid | Yes | | Yes | Yes |

| | | | | | | | | | | |
|---|---|---|---|---|---|---|---|---|---|---|
| 72 | Adenocarcinoma | 5.90 | 6.5 | LUL | | Solid | | | Yes | |
| 73 | Adenocarcinoma | 1.00 | 1.6 | RUL | | Solid | Yes | | | |
| 74 | Adenocarcinoma | 10.50 | 3.3 | LLL | | Solid | | | Yes | Yes |
| 75 | Adenocarcinoma | 6.00 | 1.6 | RUL | | Solid | | | Yes | |
| 76 | SCLC | 15.70 | 5 | RUL | Yes | Solid | | | | |
| 77 | Adenocarcinoma | 8.20 | 2 | LLL | | Solid | | | Yes | Yes |
| 78 | Adenocarcinoma | 16.50 | 5.3 | RUL | | Solid | | | | |
| 79 | Adenocarcinoma | 2.90 | 2.8 | RUL | | Mixed | | Yes | Yes | |
| 80 | Adenocarcinoma | 8.70 | 2.6 | RLL | | Mixed | | Yes | Yes | |
| 81 | Adenocarcinoma | 10.50 | 7.1 | LLL | | Solid | | | | |
| 82 | Benign lesion | 3.20 | 1.6 | LUL | | Solid | | | | |
| 83 | Benign lesion | 2.40 | 3.1 | RUL | | Solid | | | | |
| 84 | Benign lesion | 5.00 | 4.5 | RUL | | Solid | | | Yes | Yes |
| 85 | SCC | 6.70 | 3.5 | LLL | | Solid | | | | |
| 86 | SCC | 14.90 | 2 | RUL | Yes | Solid | | | Yes | |
| 87 | Adenocarcinoma | 12.60 | 3.4 | RLL | | Solid | | | | |
| 88 | Benign lesion | 5.10 | 2.1 | LUL | | Solid | | | Yes | Yes |
| 89 | SCLC | 7.40 | 5.7 | RLL | | Solid | | | | |
| 90 | Adenocarcinoma | 10.10 | 4.1 | LLL | | Solid | | | | |
| 91 | Benign lesion | 9.70 | 1.5 | RLL | | Solid | | | | Yes |
| 92 | Benign lesion | 17.70 | 4.5 | RUL | | Solid | | | | Yes |
| 93 | Benign lesion | 4.80 | 2.9 | LUL | | Mixed | | | Yes | Yes |
| 94 | Benign lesion | 1.30 | 1.5 | RUL | | Solid | | | Yes | Yes |
| 95 | SCLC | 10.80 | 7.6 | RLL | Yes | Solid | | | | |
| 96 | SCLC | 8.50 | 2.5 | LLL | | Solid | Yes | | | |

| | | | | | | | | | | |
|---|---|---|---|---|---|---|---|---|---|---|
| 97 | SCC | 5.20 | 1.2 | RLL | | Solid | | | Yes | |
| 98 | Adenocarcinoma | 6.90 | 3.8 | LUL | | Solid | Yes | | Yes | Yes |
| 99 | Adenocarcinoma | 15.20 | 5.4 | RUL | | Solid | | | | |
| 100 | Adenocarcinoma | 7.60 | 2.6 | RLL | | Solid | | | | Yes |
| 101 | Adenocarcinoma | 15.70 | 2.8 | LUL | | Solid | | | Yes | |
| 102 | Adenocarcinoma | 2.60 | 1.2 | LLL | | Solid | | | Yes | Yes |
| 103 | Adenocarcinoma | 17.20 | 3.4 | LUL | | Solid | Yes | | Yes | Yes |
| 104 | Adenocarcinoma | 4.80 | 1.7 | RUL | | Mixed | | | | |
| 105 | Benign lesion | 2.10 | 2 | RLL | | Solid | | | Yes | Yes |
| 106 | Adenocarcinoma | 9.80 | 1.1 | LUL | | Solid | | | Yes | |
| 107 | Adenocarcinoma | 4.10 | 1.9 | LLL | | Solid | | | Yes | |
| 108 | SCLC | 14.10 | 3.5 | LLL | Yes | Solid | | | | |
| 109 | Benign lesion | 0.90 | 1.2 | RLL | | Solid | | | | |
| 110 | Adenocarcinoma | 17.60 | 5.3 | RUL | Yes | Solid | | | | |
| 111 | Adenocarcinoma | 13.70 | 3.1 | LUL | | Solid | Yes | | Yes | Yes |
| 112 | Adenocarcinoma | 11.50 | 4.5 | RUL | Yes | Solid | | | | |
| 113 | Adenocarcinoma | 7.50 | 1.8 | LUL | | Solid | | Yes | | Yes |
| 114 | Adenocarcinoma | 7.40 | 1.7 | RUL | | Solid | | | Yes | Yes |
| 115 | SCC | 7.90 | 4.7 | LLL | Yes | Solid | | | | |
| 116 | Adenocarcinoma | 0.60 | 1.2 | RLL | | GGO | | | | |
| 117 | Benign lesion | 12.90 | 3.1 | RLL | | Solid | | Yes | | |
| 118 | Adenocarcinoma | 10.10 | 1.3 | LLL | Yes | Solid | | | | |
| 119 | Adenocarcinoma | 9.30 | 2 | RLL | | Solid | | | | Yes |
| 120 | SCC | 12.10 | 2.7 | LUL | Yes | Solid | | | | |
| 121 | Adenocarcinoma | 9.80 | 5.5 | LLL | Yes | Solid | | | | |

| | | | | | | | | | | |
|---|---|---|---|---|---|---|---|---|---|---|
| 122 | SCC | 26.50 | 5.7 | RLL | Yes | Solid | | | | |
| 123 | SCC | 13.90 | 4.2 | LLL | Yes | Solid | | | | |
| 124 | Adenocarcinoma | 14.90 | 6.7 | RUL | Yes | Solid | | Yes | | |
| 125 | Adenocarcinoma | 17.70 | 4.5 | RLL | | Solid | | | | |
| 126 | Adenocarcinoma | 10.60 | 1.7 | RML | | Solid | | | | |
| 127 | Adenocarcinoma | 7.30 | 6.7 | RLL | | Mixed | Yes | | | |
| 128 | Adenocarcinoma | 11.20 | 3.3 | LLL | Yes | Solid | | | Yes | Yes |
| 129 | SCLC | 10.00 | 4.9 | LLL | Yes | Solid | | | | |
| 130 | Benign lesion | 7.30 | 1.7 | LUL | | Solid | | | | Yes |
| 131 | Adenocarcinoma | 9.00 | 6.5 | RML | | Solid | | | | |
| 132 | Adenocarcinoma | 5.10 | 2.2 | LLL | | Solid | | Yes | | Yes |
| 133 | SCC | 13.70 | 9.7 | RUL | | Solid | | | | Yes |
| 134 | SCLC | 22.40 | 7.6 | Indeterminate | Yes | Solid | | | Yes | |
| 135 | SCC | 5.20 | 2.7 | RLL | | Solid | | | | Yes |
| 136 | Benign lesion | 1.60 | 1.7 | LLL | | Mixed | | Yes | Yes | Yes |
| 137 | Adenocarcinoma | 10.70 | 4.4 | RUL | | Solid | | | | Yes |
| 138 | SCC | 8.60 | 10.4 | LUL | | Mixed | | Yes | | |
| 139 | Adenocarcinoma | 7.10 | 1.3 | RUL | Yes | Solid | | | Yes | Yes |
| 140 | Adenocarcinoma | 5.10 | 1.8 | LUL | | Solid | | | Yes | Yes |
| 141 | Adenocarcinoma | 10.90 | 2.4 | RLL | | Solid | Yes | | Yes | Yes |
| 142 | Adenocarcinoma | 5.80 | 3 | RUL | | Solid | | | | |
| 143 | SCC | 19.20 | 7.2 | RLL | Yes | Solid | | | | |
| 144 | Adenocarcinoma | 9.70 | 3.5 | RLL | Yes | Solid | | | | |
| 145 | SCC | 26.60 | 2.5 | LLL | Yes | Solid | | | | |
| 146 | Adenocarcinoma | 10.10 | 11.2 | RLL | | Solid | | | | Yes |

| | | | | | | | | | | |
|---|---|---|---|---|---|---|---|---|---|---|
| 147 | Benign lesion | 10.30 | 2.2 | LLL | Yes | Solid | | | | |
| 148 | SCC | 10.20 | 4.3 | RLL | | Solid | | | Yes | |
| 149 | Adenocarcinoma | 17.20 | 4.7 | LUL | | Solid | | | | |
| 150 | Adenocarcinoma | 7.30 | 2.4 | LLL | Yes | Solid | | | | |
| 151 | Benign lesion | 3.30 | 1 | RUL | | Mixed | | | | |
| 152 | SCC | 20.40 | 2 | LLL | | Solid | | | | |
| 153 | Adenocarcinoma | 1.80 | 5.3 | RUL | | Mixed | | Yes | | |
| 154 | SCLC | 18.00 | 3.8 | RUL | Yes | Solid | | | | |
| 155 | Adenocarcinoma | 25.20 | 5.5 | RLL | | Solid | | | | |
| 156 | Adenocarcinoma | 11.70 | 5.9 | RUL | Yes | Mixed | | Yes | | |
| 157 | Benign lesion | 14.90 | 4.3 | RLL | | Solid | Yes | | Yes | |
| 158 | SCC | 20.20 | 4.7 | LLL | Yes | Solid | | | Yes | |
| 159 | SCC | 19.50 | 4.9 | RUL | Yes | Solid | | | | Yes |
| 160 | Benign lesion | 12.30 | 3.2 | RLL | Yes | Solid | Yes | Yes | | |
| 161 | SCLC | 10.80 | 2.7 | LLL | Yes | Solid | | | | |
| 162 | Benign lesion | 2.60 | 2.6 | RML | | Mixed | Yes | | | |
| 163 | SCC | 19.70 | 6.5 | LLL | Yes | Solid | | | | |
| 164 | Adenocarcinoma | 18.30 | 2.6 | RML | Yes | Solid | | | | |
| 165 | Adenocarcinoma | 8.00 | 3.3 | RUL | | Solid | | | | Yes |
| 166 | SCC | 9.12 | 6 | RLL | Yes | Solid | | | | Yes |
| 167 | SCLC | 14.50 | 3.1 | RUL | | Mixed | | | | |
| 168 | SCLC | 10.90 | Indeterminate | Indeterminate | Yes | Mixed | Yes | | | |
| 169 | SCLC | 10.40 | 4.3 | RUL | Yes | Solid | | | | |
| 170 | SCLC | 24.80 | 6.5 | RUL | Yes | Solid | | | | |

| | | | | | | | | | | |
|---|---|---|---|---|---|---|---|---|---|---|
| 171 | SCLC | 14.50 | 7.3 | RUL | Yes | Solid | | | | |
| 172 | SCLC | 9.91 | 4.2 | LUL | Yes | Solid | | | Yes | |
| 173 | SCLC | 16.90 | 3.9 | RML | Yes | Solid | | | | |
| 174 | SCLC | 15.61 | 9.1 | RUL | | Solid | | | | |
| 175 | SCLC | 3.14 | 1.5 | LLL | | Solid | | | Yes | |
| 176 | SCLC | 20.54 | 2.9 | RUL | | Solid | | | Yes | |
| 177 | SCLC | 13.40 | 4.3 | LLL | | Solid | | | Yes | |
| 178 | SCLC | 7.60 | 1.5 | LUL | | Solid | | | | |
| 179 | SCLC | 12.50 | 8.9 | LUL | Yes | Solid | | | Yes | |
| 180 | SCLC | 11.90 | 2.2 | LUL | Yes | Solid | | | | |
| 181 | SCLC | 12.00 | 5.4 | RUL | Yes | Solid | | | | |
| 182 | SCLC | 10.70 | 4.5 | Indeterminate | Yes | Solid | | | | |
| 183 | SCLC | 13.80 | 2 | RUL | Yes | Solid | | | Yes | Yes |
| 184 | SCLC | 6.50 | 2 | Indeterminate | Yes | Solid | | | | |
| 185 | SCLC | 10.50 | 5.6 | Indeterminate | Yes | Solid | | | | |
| 186 | SCLC | 10.80 | 3.3 | LUL | | Solid | | | Yes | |
| 187 | Large-cell carcinoma | 4.10 | 1.2 | RML | Yes | Solid | | | Yes | Yes |
| 188 | SCLC | 10.80 | 4.7 | LLL | Yes | Solid | | | | |
| 189 | SCLC | 9.90 | 3.9 | LLL | | Solid | | | | |
| 190 | SCLC | 11.10 | 5.6 | RLL | Yes | Solid | | | | |
| 191 | SCLC | 12.20 | 7.6 | RUL | Yes | Solid | | | | |
| 192 | SCLC | 7.90 | 11.2 | LUL | Yes | Solid | | | | |
| 193 | SCLC | 18.80 | 6.5 | Indeterminate | Yes | Solid | | | | |
| 194 | SCLC | 10.00 | 9.1 | Indeterminate | Yes | Solid | | | | |
| 195 | SCLC | 11.60 | 4.2 | RLL | Yes | Solid | | | | |

| | | | | | | | | | | |
|---|---|---|---|---|---|---|---|---|---|---|
| 196 | SCLC | 8.60 | 4.6 | RUL | | Solid | | | Yes | |
| 197 | SCLC | 16.70 | 10 | Indeterminate | Yes | Solid | | | | |
| 198 | SCLC | 15.10 | 3.7 | RUL | | Solid | | | Yes | |
| 199 | SCLC | 14.50 | 2.9 | RUL | | Solid | | | | |
| 200 | SCLC | 12.10 | 3.9 | RUL | | Solid | | | | |
| 201 | Adenocarcinoma | 3.60 | 1.7 | RUL | | Mixed | | | Yes | Yes |

RUL: right upper lobe, RML: right middle lobe, RLL: right lower lobe, LUL: left upper lobe, LLL: left lower lobe, GGO: Ground-glass opacity, SUVmax: maximal standard uptake value, SCC: squamous-cell carcinoma, SCLC: small-cell lung carcinoma.
